# Mallard resource selection trade‐offs in a heterogeneous environment during autumn and winter

**DOI:** 10.1002/ece3.4864

**Published:** 2019-02-06

**Authors:** Matthew D. Palumbo, Scott A. Petrie, Michael Schummer, Benjamin D. Rubin, Simon Bonner

**Affiliations:** ^1^ Department of Biology The University of Western Ontario London Ontario Canada; ^2^ Long Point Waterfowl and Wetlands Research Program Port Rowan Ontario Canada; ^3^ Delta Waterfowl Foundation Bismarck North Dakota; ^4^ Department of Statistical and Actuarial Sciences The University of Western Ontario London Ontario Canada

**Keywords:** anthropogenic disturbance, discrete choice, GPS satellite transmitters, Great Lakes, habitat management, hunting, Lake St. Clair, trade‐offs

## Abstract

Animals select resources to maximize fitness but associated costs and benefits are spatially and temporally variable. Differences in wetland management influence resource availability for ducks and mortality risk from duck hunting. The local distribution of the Mallard (*Anas platyrhynchos*) is affected by this resource heterogeneity and variable risk from hunting. Regional conservation strategies primarily focus on how waterfowl distributions are affected by food resources during the nonbreeding season. To test if Mallard resource selection was related to the abundance of resources, risks, or a combination, we studied resource selection of adult female Mallards during autumn and winter. We developed a digital spatial layer for Lake St. Clair, Ontario, Canada, that classified resources important to Mallards and assigned these resources a risk level based on ownership type and presumed disturbance from hunting. We monitored 59 individuals with GPS back‐pack transmitters prior to, during, and after the hunting season and used discrete choice modeling to generate diurnal and nocturnal resource selection estimates. The model that classified available resources and presumed risk best explained Mallard resource selection strategies. Resource selection varied within and among seasons. Ducks selected for federal, state and private managed wetland complexes that provided an intermediate or relatively greater amount of refuge and foraging options than public hunting areas. Across all diel periods and seasons, there was selection for federally managed marshes and private supplemental feeding refuges that prohibited hunting. Mallard resource selection demonstrated trade‐offs related to the management of mortality risk, anthropogenic disturbances, and foraging opportunities. Understanding how waterfowl respond to heterogeneous landscapes of resources and risks can inform regional conservation strategies related to waterfowl distribution during the nonbreeding season.

## 1 INTRODUCTION

Under optimal foraging theory, animals select resources by balancing the value gained from the resource and costs associated with individual body condition, risk of predation, and potential value of selecting resources elsewhere (Brown, 1988). Costs to acquire resources vary because resource quality and quantity are not distributed uniformly in space or time (Madsen, 1988; Manly, McDonald, Thomas, McDonald, & Erickson, 2002). Mortality risk is a cost that varies spatially but can be reduced by remaining in locations with decreased predation risks. However, basing resource use decisions solely upon mortality risk could compromise nutrient acquisition if these resources are of relatively poor quality, or if food availability or quality declines over time (Creel, Christianson, Liley, & Winnie, 2007; Creel, Winnie, Maxwell, Hamlin, & Creel, 2005). In highly modified landscapes, managers of private and public lands strive to conserve and augment suitable wildlife resources while also allowing wildlife‐related recreational activities (North American Waterfowl Management Plan, 2012). The balance between conserving wildlife resources and recreational opportunity must coincide with life history strategies of those species affected by management practices.

In North America, regional conservation strategies for waterfowl (Anatidae) assume that foraging resources for waterfowl during autumn and winter maybe limiting due to seasonal energetic requirements (Soulliere et al., 2007). To attract waterfowl for hunting, management practices are often aggregated into wetland complexes composed of multiple wetland types that provide a variety of resources to meet daily and seasonal needs (Dwyer, Krapu, & Janke, 1979). Wetland complexes also provide refugia or sanctuaries that vary spatially (i.e., areas free from human presence) or temporally (i.e., areas of periodic absence of human presence; Hagy et al., 2017). For example, the St. Clair National Wildlife Area prohibits hunting and restricts access. Also, hunting is not allowed within 400 m of supplemental feeding sites allowing these areas to function as spatial refugia and most private property managers restrict when hunting activities occur which creates temporal refuge for waterfowl. In contrast, public access areas are open to hunting from civil sunrise to twilight. This heterogeneity in resources and refugia can influence waterfowl movement due to the associated mortality risks from hunting while ducks meet daily nutritional needs (Cresswell, 2008; Fox & Madsen, 1997; Guillemain, Fritz, & Duncan, 2002; Madsen, 1998).

The Mallard (*Anas platyrhynchos*) is a resource generalist that is abundant in the St. Clair region. These ducks use a diversity of wetlands types and thus were a good choice to demonstrate the potential trade‐offs of mortality risk and resource selection presumed under optimal foraging theory. Harvest information suggests that the Great Lakes population of mallards could be managed separately from other midcontinent mallards due to differences in environmental conditions, available resources, and population vital rates but have been relatively less studied (Anderson & Henry, 1972; Munro & Kimball, 1982; Zuwerink, 2001). Of these vital rates, limited evidence suggests that the population of Great Lakes’ mallards may be particularly sensitive to variation in nonbreeding season survival of adult females (Coluccy et al., 2008). Nonbreeding season survival is predominately influenced by hunter harvest (Fleskes, Yee, Yarris, Miller, & Casazza, 2007; Reinecke, Schaiffer, & Delnicki, 1987), and harvest management strategies have been proposed for the Great Lakes’ mallard population (Coluccy et al., 2008). In addition to being a potentially important mortality factor, hunting influences local abundance and distribution of waterfowl (Madsen, 1998). Thus, disturbance and mortality risk associated with hunting could affect resource selection of waterfowl and have regional influences on their population dynamics. Waterfowl hunting in the Lake St. Clair region is common, and the region includes private hunt clubs, areas open to public hunting, commercial hunting guides, and waterfowl sanctuaries (Weaver et al., 2015). The spatial distribution and intensity of disturbance and mortality risk to waterfowl from hunting are variable but we presumed that the publicly accessed properties contain the greatest amount of mortality risk due the greatest use by hunters, the Canadian Wildlife Service managed property experience the least amount of mortality risk because hunting is prohibited, and the privately managed properties experience a relatively moderate amount of mortality risk because timing, location and numbers of hunters are regulated, thereby providing periods when areas are not hunted. Therefore, a better understanding of resource selection and movements of mallards within this region could influence local management practices and regional conservation of the population.

We hypothesized that resource selection was related to a combination of foraging resources and mortality risks from hunting. Therefore, we predicted resource selection models representing resource quality, quantity, and mortality risk would explain Mallard resource selection. There is variability in resource quality and associated mortality risk from hunting throughout the landscape. As such, we predicted that support for our hypothesis would be demonstrated by a greater selection for resources that have similar foraging benefits but lesser amounts of hunting activity (i.e., presumed mortality risk) demonstrating the importance of reduced risk. Additionally, we predicted that mallard resource selection for privately managed resources would have a positive influence on resource selection strategies demonstrating that mallards would expose themselves to a moderate level of risk as opposed to only selecting solely areas of spatial refugia. Our objective was to estimate Mallard resource selection based on wetland and upland cover type composition during periods when ducks were exposed to, and free from, mortality risks from hunting.

## 2 METHODS AND MATERIALS

### 2.1 Study area

**Figure 1:**
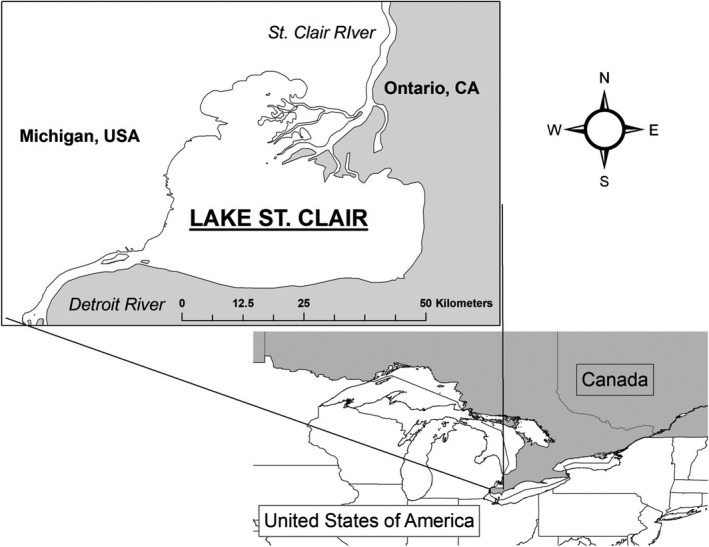
The boundaries of Lake St. Clair within the Great Lakes System

Within the Great Lakes, the Lake St. Clair region of southern Ontario, Canada, is one of the most important migratory stopovers for waterfowl (Figure 1). The area sustains thousands of waterfowl during autumn with peak dabbling duck abundance estimates of 123,000–150,000 (personal communication D. R. Luukkonen Michigan Department of Natural Resources, 9 September 2017; Dennis, North, & Ross, 1984; Weaver et al., 2015). The Canadian counties bordering Lake St. Clair (Essex, Kent, and Lambton) have experienced approximately 98% wetland loss (Ducks Unlimited Canada, 2010). Most remaining coastal wetlands are intensively managed for hunting or as inviolate waterfowl refuges. Differences in waterfowl habitat management practices and levels of human disturbance within Lake St. Clair wetland complexes provide variable foraging options and risks to waterfowl (Weaver et al., 2015). Inviolate refuges, such as the Canadian Wildlife Service National Wildlife Area (hereafter CWS‐NWA), provide roost areas of relatively low mortality risk, but food resources could potentially become limited due to greater concentrations of foraging ducks (Beatty et al., 2014a; Guillemain et al., 2002; Madsen, 1988). In contrast to refuges, public hunting areas expose birds to greater mortality risk, but may provide greater foraging opportunities due to decreased waterfowl densities. Private hunt clubs regulate hunting pressure, likely exposing birds to a moderate amount of mortality risk. However, they manage foraging resources intensively (e.g., supplemental feed and flooded agricultural crops) to offer abundant resources and to attract waterfowl.

### 2.2 Land classification data

We used land classification information from the Ducks Unlimited Canada (DUC) Hybrid Wetland Layer version 2.1.1 as our base layer for all spatial analyses of resource selection (Ducks Unlimited Canada, 2011). This digital layer contains continuous raster land cover data across Canada at a resolution of 38.7 m. To estimate property boundaries and ownership type within Ontario, we supplemented the DUC layer with spatial information that we gathered through recording property boundaries with hand held GPS units, Teranet POLARIS Boundary Data for Chatham‐Kent, the Agricultural Resource Inventory layer produced by the Ontario Ministry of Agriculture, Food, and Rural Affairs (revised 2010), spatial information from Indian Reserve layer produced by the Ontario Ministry of Natural Resources and Forestry, and GIS Open Data Website for the State of Michigan. We compiled all land classification data and property boundary data into a single spatial layer (here after, the Lake St. Clair spatial layer) through ArcMap (Environmental Systems Research Institute, Inc., Redlands, CA, USA 10.3).

### 2.3 Land class types

We measured the composition of several different land class types. We grouped the original 12 modified land classes of the DUC spatial layer into three types relevant to foraging and migrating waterfowl (agriculture, water, and marsh) and classified all other types as other. We reclassified cells as flooded agriculture based upon information from meeting landowners along the Canadian shore and identifying parcels where crops were intentionally flooded. Locations of supplemental feed in Ontario were provided by the Ontario Ministry of Natural Resources and Forestry. All raster cells within 400 m of classified feeding refuges were reclassified as a supplemental feeding refuge. Hunting was prohibited within this 400 m buffer in accordance with government permits. Therefore, after reclassification we used five land class types to represent foraging resource composition (agriculture, water, marsh, supplemental feeding refuge, and flooded agriculture; Supporting Information Table S1).

We assigned a level of hunting intensity and risk to each resource type, based on ownership type because property managers regulate the frequency and duration of hunting activities (daily and seasonally). Public property had the fewest restrictions to hunter access, frequency, and hours afield. The most restrictive ownership type was the CWS‐NWA that prohibited hunting. Property types of private, Walpole Island, and MICH‐DNR were assumed to be at an intermediate risk level because these properties allow hunting but manage the frequency, location, and duration. Hunting is prohibited within the 400 m of supplemental feeding sites, but they are located within private property boundaries with the management goal of attracting waterfowl to be harvested. Therefore, we assigned the level of risk associated with using a supplemental feeding refuge as intermediate relative to other resource types. Overall, our most detailed land classification represented the combination of resources and ownership type (Table 1). We did not have similar habitat information for MICH‐DNR as we did for the DUC layer and only categorized MICH‐DNR as a different ownership type.

**Table 1:** List of variables, variable abbreviations for model specification, variable description, and available area used for all resource selection models of Mallards in the Lake St. Clair region during autumn and winter of 2014–2015 and 2015–2016

Variable	Variable abbreviation	Variable description	Area (ha)
Michigan St. Clair Flats	MICH‐DNR	Area of property managed by the Michigan Department of Natural Resources within the St. Clair Flats	4548.95
Public Water	PUB‐WATER	Area of water in Lake St. Clair on the Ontario side that is accessible to the public.	77796.36
Private Water	PRI‐WATER	Area of water under private management in southwestern Ontario	9904.84
Walpole Island Water	WAL‐WATER	Area of water under Walpole Island management	1325.88
Michigan Water	MICH‐WATER	Area of Lake St. Clair that is on Michigan side of the lake	27759.99
Public Marsh	PUB‐MARSH	Area of marsh in Lake St. Clair that is accessible to the public in Ontario	201.55
Private Marsh	PRI‐MARSH	Area of marsh under private management in southwestern Ontario	2448.56
Walpole Island Marsh	WAL‐MARSH	Area of marsh under Walpole Island management	6307.78
Federal Marsh	CWS‐MARSH	Area of marsh under management of the Canadian Wildlife Service	308.40
Federal Water	CWS‐WATER	Area of water under management of the Canadian Wildlife Service	20.26
Private Flooded Agriculture	PRI‐FLAG	Area of flooded agriculture under private management in southwestern Ontario	167.93
Private Supplemental Feed	PRI‐SUPP	Area of supplemental feed under private management in southwestern Ontario	926.54
Private Agriculture	PRI‐AGRI	Area of dry agriculture under private management in southwestern Ontario	161110.09
Walpole Island Agriculture	WAL‐AGRI	Area of dry agriculture under Walpole Island management	3899.30

### 2.4 Capture and transmitter deployment

In late August and early September of 2014 and 2015, we captured and marked ducks (*n = *59) on private property along the Canadian shore of Lake St. Clair (UTM 17 N 383701 E, 4697376 N), using a swim‐in trap baited with shelled corn. We determined age as hatch‐year (a duck that hatched that calendar year) or after‐hatch‐year (a duck that hatched before that calendar year; hereafter adult) based on wing plumage and retrices (Carney, 1992). We determined sex based on wing coloration and cloacal examination. We inspected wing plumage to determine whether ducks had finished molting for transmitter attachment. Of the 2014 cohort (*n = *20 ducks), nine adult female Mallards were equipped with 22 g Platform Terminal Transmitters (PTT, NorthStar Science and Technology, LLC, King George, Virginia, USA) back‐pack style solar powered Global Positioning System (GPS) transmitters (Model 22GPS). The remaining 11 were equipped with 25 g GSM back‐pack style GPS transmitters (Model Saker‐H, NorthStar Science and Technology, LLC, King George, Virginia, USA and Ecotone Telemetry, Sopot, Poland). PTTs were programed to collect six fixes per 24 hr period while the GSM transmitters were programed to collect eight fixes per 24 hr period. We used a combination of transmitters initially because we did not know how the cellular network of the study area would affect GSM transmitter performance. The GSM transmitters from the 2014 cohort performed adequately, therefore due to their greater fix rate and lower cost, the entire 2015 cohort (*n* = 39) consisted of 25 g GSM back‐pack style GPS transmitters.

Transmitters were equipped with a 3.5 g Very High Frequency (VHF) transmitter (Holohil Systems Ltd., Carp, ON, Canada) enabling us to determine fate and transmitter status. We trimmed and glued a 3.2 mm neoprene pad to the base of each transmitter and attached transmitters dorsally between the wings using a harness of 0.38 cm wide Teflon ribbon (Bally Ribbon, Bally PA, USA). The completed harness was one continuous strand of ribbon that included posterior and anterior body loops knotted to connect over the keel (Krementz, Asante, & Naylor, 2011, 2012; Petrie, Rogers, & Baloyi, 1996). Total transmitter package weight was ≤32 g and was ≤5% of the body mass of marked ducks (average body mass at capture was 1072.05 ± 21.26 g) as recommended by the guidelines for transmitter mass by the American Ornithologists Union (Fair et al., 2010). Ducks were released immediately after being equipped with GPS transmitters (Animal Use Protocol 2014–017).

### 2.5 Temporal scale

We censored the first 4 days of GPS fixes to allow ducks to recover from handling and transmitter attachment (Cox & Afton, 1998). We used legal shooting time to categorize the period of GPS fixes as diurnal (if it occurred from 30 min before sunrise to 30 min after sunset) or nocturnal (fixes outside of this time). We monitored ducks until 31 January, the transmitter failed to report fixes, or a duck was reported shot by a hunter (Supporting Information Appendices S1 and S2). GPS fixes from both monitoring years were combined to increase sample size, and we then divided the study data into four seasons to examine differences in resources selection over time. Seasons were based on the 106‐day Ontario southern district open hunting season for ducks; PRE‐hunting season (27 August 2014–26 September 2014 and 30 August 2015–25 September 2015); FIRST half of the hunting season (27 September 2014–18 November 2014 and 26 September 2015–17 November 2015); SECOND half of the hunting season (19 November 2014–10 January 2015 and 18 November 2015–9 January 2016); and POST‐hunting season (11 January 2015–31 January 2015 and 10 January 2016–31 January 2016). We divided the hunting season into early and late periods because food availability, thermoregulatory costs, waterfowl abundance, and hunting pressure change during the 106‐day waterfowl season. There was no legal hunting during the PRE and POST‐hunting seasons.

### 2.6 Spatial scale

We categorized step lengths (i.e., distance between GPS fixes) that were >0.33 km but <25 km as local movements. We considered any step length <0.33 km as a fine scale movement and anything >25 km as a relocation movement. Our categorized range of local movements was similar to recently published movements for dabbling ducks (0.25–30.0 km; Beatty et al., 2014b; Davis & Afton, 2010; Jorde, Krapu, & Crawford, 1983; Link, Afton, Cox, & Davis, 2011; Supporting Information Appendix S3, Figure S1). We used local scale fixes for statistical analysis because movements within this range could be influenced by resource components similar to 3rd order selection (Johnson, 1980), and our land classification data represented these components.

### 2.7 Statistical analysis

#### 2.7.1 Identifying choice sets

**Figure 2:**
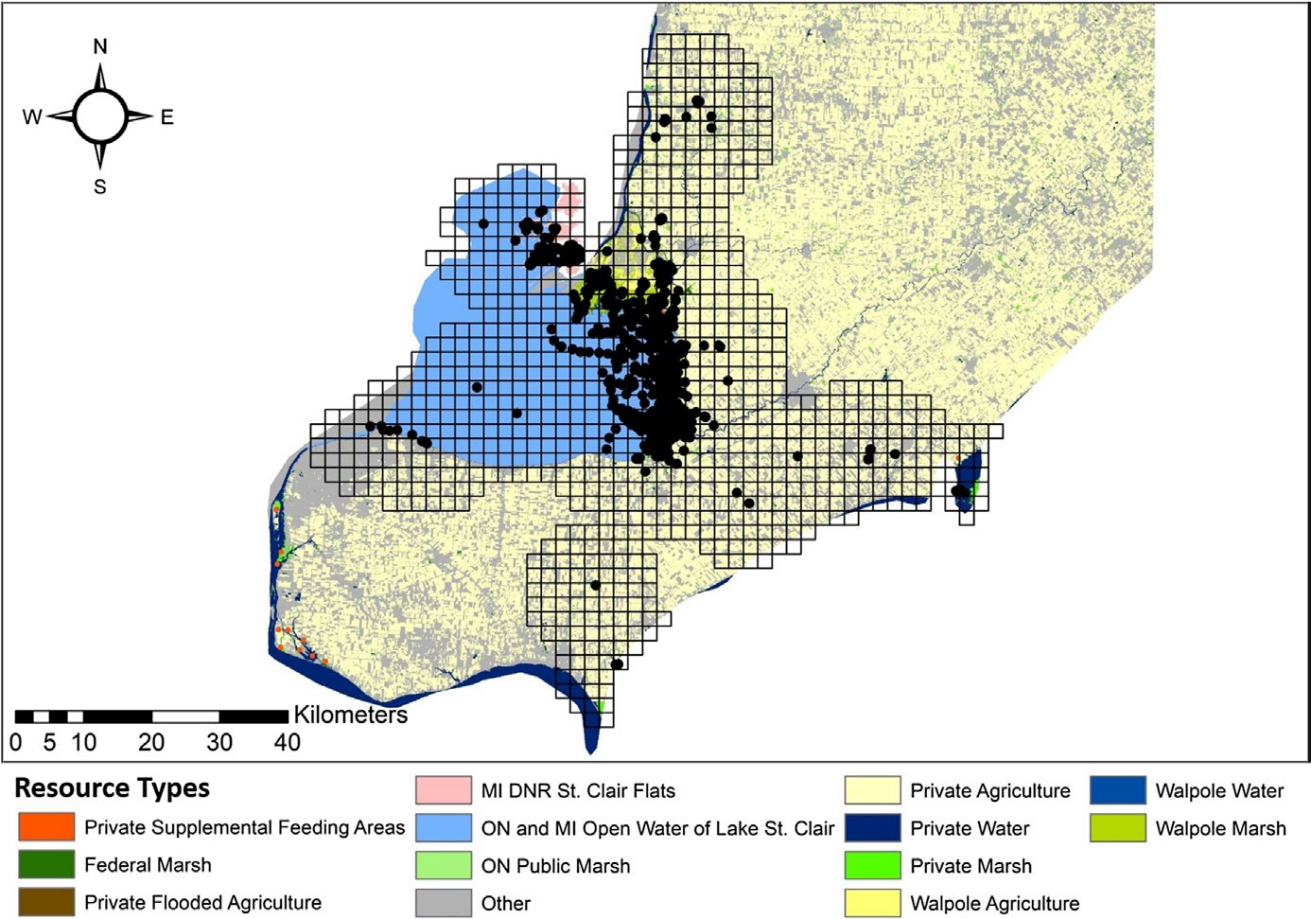
The GPS fixes of the local movements and the grid cells of all resource units used to determine adult female Mallard resource selection within the Lake St. Clair region

We used discrete choice models to investigate local scale (movements 0.33–25.0 km) resource selection in the Lake St. Clair region (Beatty et al., 2014b; Cooper & Millspaugh, 1999; Thomas, Johnson, & Griffith, 2006). Total sample size was the number of choice sets, where in each choice set, one used resource unit was selected from a group of available resource units (Cooper & Millspaugh, 1999; McCracken, Manly, & Heyden, 1998). To discretely categorize resource units, we over laid local scale GPS fixes with a grid system of 2.12 km^2^ cells across the Lake St. Clair spatial layer using Geospatial Modeling Environment Version 07.4.0 (Beyer, 2015) and ArcMap (Environmental Systems Research Institute, Inc., Redlands, CA, USA 10.3. 1.; Carter, Brown, Etter, & Visser, 2010; Thomas et al., 2006). We determined grid cell area from the average step length for all local scale movements (Beatty et al., 2014b). We intersected local scale GPS fixes with the grid system, and grid cells that contained a GPS fix were categorized as a used resource unit. Choice sets included available resource units that were grid cells whose center was within 9.6 km from the center of the used resource unit (Figure 2). The radius of 9.6 km represented the 97.5th quantile of all step lengths within the local scale movements (Güthlin et al., 2011) We estimated the area (ha) of variables within in each resource unit using ArcMap (Environmental Systems Research Institute, Inc., Redlands, CA, USA 10.3. 1) and Geospatial Modeling Environment Version 07.4.0 (Beyer, 2015).

#### 2.7.2 Discrete choice models

We used a Bayesian random‐effects multinomial logit model, (i.e., mixed logit discrete choice model), that incorporates each individual as a random‐effect to account for correlation from repeated observations (Beatty et al., 2014b; Thomas et al., 2006). Bayesian random‐effects models allow for estimating individual and population‐level selection coefficients given the observed data (i.e., GPS fixes). We used the modeling approach and discrete choice equation developed by Beatty et al. (2014b), Supporting Information Appendix S4). We used all possible alternatives in a choice set but the number of alternatives within a choice set varied depending on the location of the used resource unit and the edge of the Lake St. Clair spatial layer. The maximum size of choice set consisted of 69 resource units.

We assumed that all individual‐level coefficients of all independent variables were normally distributed with population mean centered at zero and standard deviation *σ_k_* to generate population‐level coefficients. For all hyper‐parameters, we assumed prior distributions with *μ_k_* ~ *Normal*(0, 2.786), following Newman (2003) and *σ_k_* ~ *t*(0, 2, 3) truncated to remain positive, as recommend by Gelman (2006). We also detected that these priors assisted with achieving model convergence. To construct discrete choice models, we identified the independent variables whose area estimates within choice sets that were not highly correlated (pair‐wise |*r*| < 0.8) using the Pearson correlation matrix for each season and each diel period (Staub, Binford, & Stevens, 2013). This process reduced convergence issues with multicollineartiy but retained variables of biological interest. We categorized each diel period (day, night) as a subset of our data for each season (PRE, FIRST half of the hunting season, SECOND half of the hunting season, POST) for a total of 32 separate models (four seasons × two diel periods × four candidate models; Supporting Information Table S2). Each model represented a biological hypothesis that Mallard resource selection was related to either resource abundance, mortality risk, a combination of resource abundance and mortality risk, or was random (Supporting Information Table S2). We ranked the four candidate models by their deviance information criterion (DIC), the Bayesian analog to Akaike's information criterion (Beatty et al., 2014b; Burnham & Anderson, 2002; Spiegelhalter, Best, Carlin, & van der Linde, 2002). We calculated ΔDIC values from the top most parsimonious model and used >5 ΔDIC units to determine which models were competitive for each diel period and season (Beatty et al., 2014b; Thomas et al., 2006). We were specifically interested in population‐level resource selection strategies; thus, we based inferences on the posterior distribution of the population‐level mean *μ_k_* and its 95% credible intervals for each top‐ranking model (Beatty et al., 2014b). We further inferred that variables whose 95% credible intervals that did not include zero as being important in the resource selection models (Beatty et al., 2014b). We fitted candidate discrete choice models in JAGS v 4.2.0 using the package R2jags (Su & Masanao, 2015) in R version 3.3.2 (R Development Core Team, 2016). We used the function jags.parallel within this package to run three separate chains for all candidate models. The number of iterations, thinning, and burn‐in varied per season and candidate model (Supporting Information Table S3). We used Brooks–Gelman–Rubin statistic as an assessment of convergence where values <1.1 indicate convergence to the posterior distribution (Brooks & Gelman, 1998; Gelman & Hill, 2007). We standardized all independent variables using two standard deviations $\left(\frac{x_{\text{i}}\;\text{-}\;\mu_{\text{x}}}{2S_{\text{x}}}\right)$ to interpret coefficients on a common scale (Beatty et al., 2014b; Gelman & Hill, 2007).

## 3 RESULTS

We censored two ducks during the first 4 days of monitoring leaving 57 to study Mallard resource selection. We used 42,273 GPS fixes to calculate movement distances. To isolate the local scale movements for resource selection analysis, we removed 30,571 fine scale movements and 100 relocation scale movements, resulting in 11,602 local scale movements. Of the local scale movements, we removed 1,447 fixes that were beyond the extent of geospatial data. Therefore, our final sample included 10,155 GPS fixes. The number of individuals per season and diel period ranged from 19 to 57, and the total number of fixes per season and diel period varied from 199 to 2,191 (Table 2). We did not track individual ducks for more than 1 year.

**Table 2:** Descriptive statistics of adult female Mallard GPS transmitter data during 2014–2015, and 2015–2016 monitoring years, including season, diel period, number of individuals (*IDs*), sum of fixes (*N*), mean fixes per individual ($\overset{¯}{X}$), standard deviation (*SD*), and range of fixes per individual, that were used for resource selection analyses

Season	Diel period	*IDs*	*N*	$\overset{¯}{X}$	*SD*	Range
PRE	Diurnal	57	1724	30.25	13.86	2–59
	Nocturnal	56	771	13.77	7.97	1–35
FIRST	Diurnal	51	2,191	42.96	24.76	1–99
	Nocturnal	50	1,895	37.9	21.03	1–76
SECOND	Diurnal	42	1,550	36.9	18.19	1–73
	Nocturnal	41	1,583	38.61	18.22	1–81
POST	Diurnal	19	242	12.74	7.86	1–26
	Nocturnal	19	199	10.47	7.09	2–27

**Figure 3:**
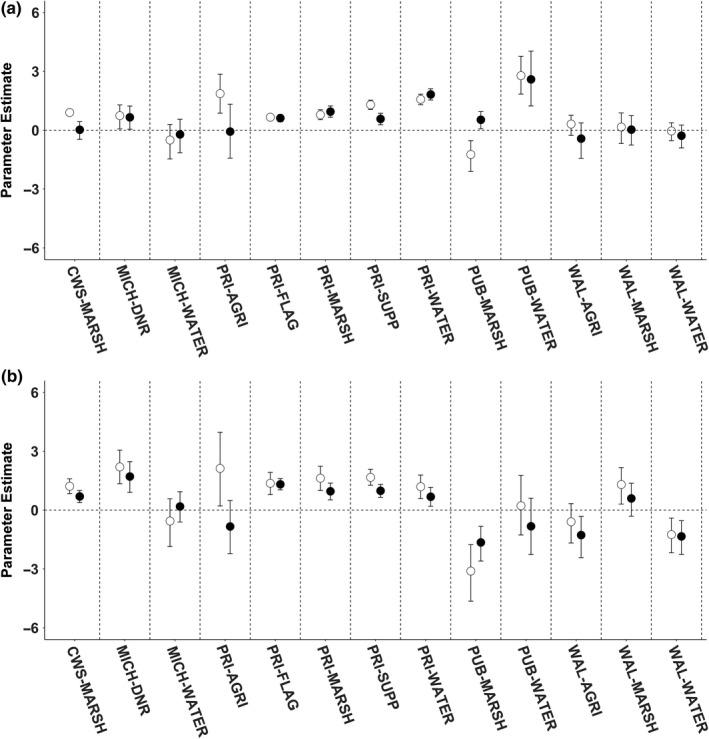
Parameter coefficients and 95% credible intervals for the top‐ranking discrete choice models that investigated resource selection strategies for adult female Mallards during the PRE‐hunting season (a) and during the FIRST half of the hunting season (b), in the Lake St. Clair region during the 2014–2015 and 2015–2016 monitoring periods. White circles represent parameter estimates of diurnal models, and black circles represent parameter estimates of nocturnal models

**Figure 4:**
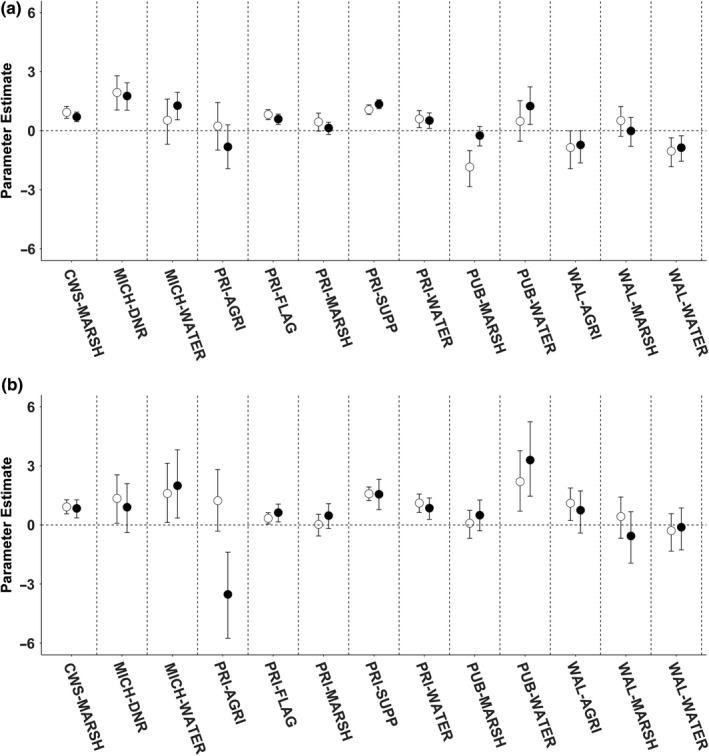
Parameter coefficients and 95% credible intervals for the top‐ranking discrete choice models that investigated resource selection strategies for adult female Mallards during the SECOND half of the hunting season (a) and during the POST‐hunting season (b), in the Lake St. Clair region during the 2014–2015 and 2015–2016 monitoring periods. White circles represent parameter estimates of diurnal models, and black circles represent parameter estimates of nocturnal models

Based on the Pearson correlation matrix, we removed the CWS‐WATER variable as its occurrence in choice sets was highly correlated (*r* > 0.8) with CWS‐MARSH. The top model for every season and diel period was the full model that categorized resource units by area of resource type and ownership type representing a combination of resource abundance and mortality risk (Table 3, Supporting Information Table S4). Influential resource selection parameters were variable per season and diel period. During the PRE‐season, adult female Mallards selection was positively influenced by the area of federally managed marsh and private agriculture during the daytime only. Ducks also selected for MICH‐DNR, private flooded agriculture, private marsh, private supplemental feeding, private water, and public water during both diel periods. Public marsh was avoided during the day and selected for at night. The posterior distribution for all other variables overlapped zero (Figure 3, Supporting Information Table S5). During the FIRST half of the hunting season, ducks began to select federally managed marsh at night, avoiding public marsh during day and night, and the shift in the posterior distribution of public water to include zero suggesting that the influence of this variable was not substantial. Ducks also began to select for Walpole Island marsh during the day while avoiding Walpole Island water and agriculture at night (Figure 3, Supporting Information Table S6). During the SECOND half of the hunting season, ducks selected public water and Michigan water at night. Many of the other landscape composition variables continued to be selected by ducks but the posterior distributions of private agriculture and private marsh, and Walpole Island marsh overlapped zero (Figure 4, Supporting Information Table S7). During the POST‐season, adult female Mallards selected federally managed marsh, Michigan water, private flooded agriculture, supplemental feeding refuges, private water, and public water. During the day, Ducks selected MICH‐DNR and Walpole Island agriculture while avoiding private agriculture at night. The posterior distribution of all other landscape composition variables included zero (Figure 4, Supporting Information Table S8).

**Table 3:** Delta deviance information criterion values for all resource selection models during the PRE‐hunting season, FIRST half of the hunting season, SECOND half of the hunting season, and POSTߚhunting season seasons and for both diurnal and nocturnal diel periods

DELTA deviance information criterion values								
Model	PRE		FIRST		SECOND		POST	
	Diurnal	Nocturnal	Diurnal	Nocturnal	Diurnal	Nocturnal	Diurnal	Nocturnal
4	0.0	0.0	0.0	0.0	0.0	0.0	0.0	0.0
3	1638.3	1658.0	1530.9	1267.6	746.0	514.0	62.1	109.9
2	4079.5	2171.0	4687.5	4389.1	2877.6	3632.9	344.8	392.7
1	6922.2	2852.9	13680.6	10456.5	7819.6	6751.2	770.7	695.8

## 4 DISCUSSION

A key component to conservation is the consideration of how resource selection is influenced by landscape heterogeneity and anthropogenic disturbances (Beatty et al., 2014a). Conservation and management of wetland complexes are conducted by stakeholders that use various strategies to maximize use, productivity, biodiversity, and to sustain ecological services (Euliss, Smith, Wilcox, & Browne, 2008). Therefore, it is valuable for natural resource managers to understand how animals select resources given the diversity of available resources, disturbances, and mortality risks. The results of our modeling process support our hypothesis and predication that Mallard resource selection was related to a combination of resource quality, quantity, and mortality risks from hunting. Herein, we discuss the parameter estimates of different variables in our top‐ranked models that exemplify how ducks were balancing daily and seasonal trade‐offs between resources and mortality risk.

During the hunting season, Mallards decreased selection for public water which we presumed experienced the greatest use by hunters. In contrast, waterfowl continued to select private water throughout the hunting season. Based on the digital classification of resource types, private and public water had similar foraging resources but different mortality risks from hunting (Palumbo, 2017). Therefore, we suggest that ducks were selecting private water during the hunting season to benefit from reduced hunting pressure, relative to public water (Dooley, Sanders, & Doherty, 2010a, 2010b). Also, private marsh and CWS‐NWA (i.e., federally managed marsh) provided similar foraging benefits with different levels of mortality risk. We detected that there was no longer a substantial influence of private marsh on selection after the FIRST half of the hunting season. We suggest this response was related to a shift in the balance of trade‐offs, because ducks continued to select for CWS‐NWA, a similar vegetation type, but with no mortality risk from hunting (Dooley, Sanders, & Doherty, 2010a, 2010b; Madsen 1988). Disturbances and risks at private marshes may have had a chronic effect on Mallard distribution, making the cost associated with using this wetland type outweigh the benefit over time.

Wetland managers on private lands flood unharvested agricultural fields to provide waterfowl foraging opportunities and roosting sites. Despite being hunted and therefore representing a substantial mortality risk, flooded agricultural fields were selected by Mallards both diurnally and nocturnally and throughout the monitoring period (Figures 3 and 4) suggesting that ducks were able to navigate the perceived hunting‐related mortality risks to access resource benefits. Our results suggest that flooded agricultural fields provide energy dense foods and roosting habitat, similar to other locations (Pearse, Kaminski, Reinecke, & Dinsmore, 2012). Field‐feeding waterfowl generally increase time spent foraging in agricultural fields as weather conditions deteriorate to balance thermoregulatory costs and prepare for migration (Jorde et al., 1983). However, field‐feeding waterfowl can quickly deplete food availability in fields, (Foster, Gray, & Kaminski, 2010; Hagy & Kaminski, 2015) and postharvest treatments and snow cover can substantially reduce accessibility of waste grains (Baldassarre & Bolen, 1984; Schummer, Kaminski, Raedeke, & Graber, 2010; Stafford, Kaminski, & Reinecke, 2010). Ducks in our study decreasingly used fields as the season progressed suggesting the relative benefit of selecting for agricultural fields decreased over time.

Wetland managers provide supplemental feeding refuges to attract and hold ducks at their wetland complexes but are prohibited from hunting within 400 m of the deposit site. To access these refuges that contain a substantial foraging and safety benefit, ducks are exposed to a relatively moderate energetic and mortality cost of traveling over locations that are hunted during the day. The dense abundance of corn at the deposit site and variable amounts of other vegetation types allow ducks to perform their daily activities (i.e., foraging, thermoregulation, and courtship) without needing to relocate to other spatial patches where hunting pressure can be substantial. Areas that prohibit hunting are prioritized as critical to waterfowl conservation (Beatty et al., 2014a; Madsen, 1998), but can be difficult to incorporate in regional conservation strategies due to variability in implementation. For example, different hunting season durations in Michigan, variable hunting strategies on Walpole Island, and variable hunting management on private property limited our estimate of spatial refuge. Despite this limitation, consistent selection by Mallards for refuge areas, including supplemental feeding areas, suggests benefits derived from them were important to waterfowl in the region.

The Lake St. Clair region is characteristic of many areas that have experienced wetland loss with the majority of wetland management occurring on private lands adjacent to government managed complexes. Our resource selection analysis supports the importance of areas protected from anthropogenic disturbance within wetland complexes (Beatty et al., 2014a), in addition to providing foraging resources, during autumn and winter. In addition to determining foraging resources, regional conservation strategies could benefit from incorporating how waterfowl distributions are influenced by variable risks, similar to the relative ranking in our study. Furthermore, understanding how these management practices influence survival would provide an estimate of how management practices are linked to the fitness trade‐offs we described.

## CONFLICT OF INTEREST

None declared.

## AUTHOR CONTRIBUTIONS

MP, SP, and MS conceived the study, designed methodology, and acquired funds. MP collected the data. MP, SB, and BR analyzed the data. MP led the writing with substantial editorial guidance from SP and MS. All authors made substantial contributions to previous drafts and have approved the final version.

## Data Availability

We intend to archive our data in the Dryad Digital Repository.
