# Supplementary material for: Mallard resource selection trade‐offs in a heterogeneous environment during autumn and winter
Source: Ecol Evol. 2019 Feb 6;9(4):1798–808. doi: 10.1002/ece3.4864 (PMC6392399; doi:10.1002/ece3.4864)
Supplement: Supplementary file 1 [file ECE3-9-1798-s001.docx]

Supporting-Table 1. The categorized vegetation types from the DUC 2011 Hybrid Wetland Layer Version 2.1.1 and their associated variable names.

^a^Vegetation types classified as other were not used in the resource selection analysis.

|  | | | |
| --- | --- | --- | --- |
| Data Source | Vegetation Type | Categorized Vegetation type | Variable Name |
| Ducks Unlimited Hybrid Wetland Layer 2.1.1 | Cropland | Agriculture | AGRI |
|  | Agriculture |  |  |
|  | Water | Water | WATER |
|  | Wetland | Wetland | MARSH |
|  | Upland | Other | NA^a^ |
|  | Non-vegetated |  |  |
|  | Developed |  |  |
|  | Shrubland |  |  |
|  | Native Grassland |  |  |
|  | Forage/Pasture/Perennial Crops |  |  |
|  | Coniferous |  |  |
|  | Broadleaf |  |  |
|  | Mixedwood |  |  |
| Landowners | Flooded Agriculture | Flooded Agriculture | FLAG |
| Ontario Ministry of Natural Resources and Forestry | Supplemental Feeding Areas | Supplemental Feeding Areas | SUPP |

Supporting Information 1. Temporal Scale Information

We converted all time data to Eastern Standard Time or Eastern Daylight Savings time and determined the time of sunset and sunrise for each fix from National Oceanic and Atmospheric Administration (NOAA) Earth System Research Laboratory (ESRL) Global Monitoring Division daily solar calculation spread sheet using the approximate center of St. Clair National Wildlife Area St. Clair Unit as the reference location (<http://www.esrl.noaa.gov/gmd/grad/solcalc/calcdetails.html>).

Supporting Information 2. Criteria used to determine what GPS fixes to use when transmitters were recovered.

Within the Lake St. Clair spatial layer, if transmitters failed to report fixes for multiple duty cycles, we attempted to recover carcasses and transmitters by searching at the last known GPS location through homing to the VHF signal using a VHF receiver and a hand-held Yagi antenna .We monitored individual duck locations by downloading GPS fixes from manufacturer provided software and internet access portals. If ducks did not move approximately >150 m between GPS fixes we waited until GPS fixes were no longer being transmitted for multiple duty cycles before attempting to determine the fate of the individual. We implemented this strategy because the unsuccessful transmission of GPS fixes could have been a result of poor reception between the transmitter and the Argos satellites or GSM network and not an indication of a mortality event. Also, because access to many of the locations the ducks used was limited, and a goal was to not introduce extra disturbance.

The GPS transmitters were programmed to store thousands of GPS points until a suitable connection to off-load fixes was established. When we could recover the GPS unit and recharge the solar battery there was the possibility of downloading more GPS fixes prior to what was known at the time of recovery. Thus, criteria for determining when GPS fixes stopped being representative of a live duck were based on a set of scenarios:

(1) if we recovered the carcass with an attached transmitter at a location that was ≤ 150 m from the location of the last reported GPS fix, then the date the bird arrived at that location and GPS fixes moved <150 m was the date of death and the last location used;

(2) if we recovered the carcass with an attached transmitter >150 m from the last reported location, we used all of recorded GPS fixes and randomly picked a day from the last day a fix was transmitted to the day before we found the transmitter as the date of death (Frair, Merrill, Allen, & Boyce, 2007);

(3) if GPS fixes stopped being transmitted and we could not recover the transmitter the last downloaded fix was used as the last location for that bird.

(4) for harvest mortalities, we considered the duck to have been alive and provided accurate location information prior to the time when the hunter reported harvesting the bird.

Frair, J. L., Merrill, E. H., Allen, J. R., & Boyce, M. S. (2007). Know thy enemy: experience affects elk translocation success in risky landscape. Journal of Wildlife Management, 71, 541–554.

Supporting Information 3. Spatial Scale Information

We restricted the spatial extent of our study to southwestern Ontario and MICH-DNR. To determine the scale of resource selection within this region, and define the size of resource units, we used movement data from all marked ducks (Boyce, 2006). We examined the movement patterns of individuals by calculating the distance between GPS fixes (i.e., step lengths) using ArcMET (Movement Ecology Tools for ArcGIS, version 10.3.1 v1) through ArcMap (Environmental Systems Research Institute, Inc., Redlands, CA, USA 10.3. 1.). To decrease the influence of movements that happened when transmitter and satellite connectivity was substantially less than the programmed duty cycle, we only used intervals that were < 24 hrs apart (Beatty et al., 2014). Also, to decrease the effects of GPS fixes downloaded in errant rapid succession outside of the programmed duty cycles, we only used GPS fixes that were > 2 hrs apart. We calculated the natural log transformation of all step lengths > 0 km to plot the observed distribution of movement distances. We fitted a Gaussian kernel density estimator to the natural log transformed observed distribution using the geom_density function in the ggplot2 package (Wickham & Winston, 2016) of R version 3.3.2 (Beatty et al., 2014; R Development Core Team, 2016).

We classified each GPS fix into one of three spatial groupings based on the straight-line distance from the preceding fix. We partitioned spatial scale categories based on visually identifying breaks in the distribution of the smoothed data (Beatty et al., 2014).

Supp-Fig 1. Spatial scales based on the probability density of natural log transformed step lengths for adult female Mallards during the 2014–15 and 2015–16 monitoring periods. Distance moved corresponds to the natural log of the distance between GPS focal fix *a* and the previous fix *a* – 1, for focal fix *a*. Transformed distances in kilometers are on the x axis.


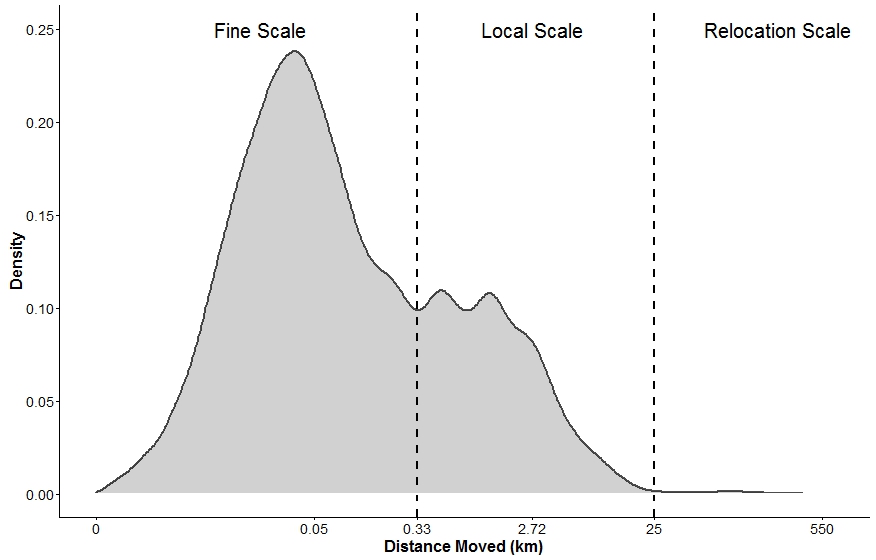


Beatty, W. S., Webb, E. B., Kesler, D .C,. Raedeke, A. H., Naylor, L. W., & Humburg, D. D. (2014). Landscape effects on Mallard habitat selection at multiple spatial scales during the non-breeding period. Landscape Ecology, 29, 989–1000. doi:10.1007/s10980-014-0035-x

Boyce, M. S. (2006). Scale for resource selection functions. Diversity and Distributions 12, 269–276. doi: 10.1111/j.1366-9516.2006.00243.x

Wickham, H., & Winston, C. (2016) ggplot2: Create elegant data visualisations using the grammar of graphics. R package version 2.2.1

Supporting Information 4. Multinomial logit hierarchical model variables and JAGS model

Multinomial logit hierarchical model in JAGS to examine resource selection of adult female mallards in the Lake St. Clair region, models were run using R2jags. Modeling procedure was adjusted based on modeling procedure from Beatty et al. 2014*.* Data were initially in long format where one row represents one alternative within a choice set. Each choice set contains as many rows as there are alternatives.

**Variables:**

T = the total number of rows in the data sheet.

chsets = indexes choice sets in long format, ranges from 1 to the total sample size.

alts = indexes alternatives in long format within a choice set, maximum range is 1 69.

df.3 = orders the number of alternatives in each choice set.

nalts = the number of alternatives within a choice set with a maximum of 69.

use = use in long format, 0 for available resource units, 1 for used resource units.

nchsets = the number of choice sets, equal to the total sample size.

DuckID = indexes individual ducks in long format, ranges from 1 to ninds.ID

ninds = the number of unique individuals.

cws_marsh_stand = An example of one variable used in the model. cws_marsh_stand is area (hectares) of federal marsh with 2.12 km^2^ resource unit, centered and standardized.

pri_agri_stand = An example of one variable used in the model. pri_agri_stand is area (hectares) of private agriculture within 2.12. km^2^ resource unit, centered and standardized.

X = a matrix of habitat variables in long format.

npred = the number of predictors, equal to the number of columns for matrix X.

duckid2 = indexes individual ducks in wide format, ranges from 1 to ninds.

y = matrix of used and maximun available resource units

first.day.1 <- read.csv("first.day.1.csv")

T <- nrow(first.day.1)

chsets <- first.day.1$chsets

alts<- first.day.1$index

df.3 <- summarize(group_by(first.day.1, chsets), n.alt3 = max(index))

nalts<-df.3$n.alt3

use <- first.day.1$choice.x

nchsets <- max(first.day.1$chsets)

ninds <-max(first.day.1$DuckID)

cws_marsh_stand <- first.day.1$cws_marsh_stand

pri_agri_stand <- second.night.1$pri_agri_stand

X = cbind(cws_marsh_stand, pri_agri_stand)

npred <- ncol(X)

duckid2 <- subset(first.day.1, choice.x==1)$DuckID

y=cbind(1,matrix(0,nrow=nchsets,ncol=nalts-1))

jags.data <- list(npred=as.integer(npred),

duckid2=as.integer(duckid2),

ninds=as.integer(ninds),

nchsets=as.integer(nchsets),

nalts=as.integer(nalts),

y=cbind(1,matrix(0,nrow=nchsets,ncol=nalts-1)))

#Transcribe X matrix into wide format per number of alternatives

jags.data$Z <- array(NA,dim=c(npred,nchsets,max(nalts)))

for (i in 1:T) {

for (j in 1:npred) {

jags.data$Z[j,chsets[i],alts[i]] <- X[i,j]}

}

**JAGS Model:**

model {

## Priors

for (a in 1:ninds){

for (j in 1:npred) {

beta[a,j] ~ dnorm(0, tau[j])

}

}

## Hyperparameters

for (j in 1:npred){

mu[j] ~ dnorm (0,.359)

sig[j] ~ dt(0,2,3)T(0,)

tau[j] <- 1/sqrt(sig[j])

}

## Likelihood

for (i in 1:nchsets) {

y[i,1:nalts[i]] ~ dmulti(p[i,1:nalts[i]], 1)

for (k in 1:nalts[i]) {

log(phi[i,k]) <- inprod(mu[] + beta[duckid2[i],],Z[,i,k])

p[i,k] <- phi[i,k] / sum(phi[i,1:nalts[i]])

}

}

}

", fill = TRUE)

Beatty, W. S., Webb, E. B., Kesler, D .C,. Raedeke, A. H., Naylor, L. W., & Humburg, D. D. (2014). Landscape effects on Mallard habitat selection at multiple spatial scales during the non-breeding period. Landscape Ecology, 29, 989–1000. doi:10.1007/s10980-014-0035-x.

Supporting-Table 2. List of Candidate models and variables representing adult female mallard resource selection in the Lake St. Clair regions during the 2014–15 and 2015–16 monitoring periods. Please see Table 1 for variable definitions.

| Model Number | Model |
| --- | --- |
| 1 | (NULL) |
| 2 | (PUB)+(PRI)+(WAL)+(CWS)+(MICH-DNR) |
| 3 | (WATER)+(MARSH)+(FLAG)+(SUPP)+(AGRI)+(MICH-DNR) |
| 4 | (PUB-WATER)+(PRI-WATER)+(WAL-WATER)+ (CWS-WATER)+(MICH-WATER+(PUB-MARSH)+(PRI-MARSH)+(CWS-MARSH)+(WAL-MARSH)+(PRI-FLAG)+(PRI-SUPP)+ (PRI-AGRI)+(WAL-AGRI)+(MICH-DNR) |

Biological hypotheses for each candidate model.

Model (1) was a null model that assumed the probability of use for all alternatives within a choice set was equal to random chance.

Model (2) was an ownership model that included resource types grouped by ownership as the dependent variables. This model would be appropriate if waterfowl resource selection was influenced by the management practices of the property owner (i.e., amount of disturbance and mortality risk from hunting) but not the composition of the vegetation.

Model (3) was a landscape composition model that includes the area of different vegetation types within each resource unit. I included the St. Clair Flats as a single vegetation type as I did not have access to similar spatial data that were available for southwestern Ontario. This model would be appropriate if waterfowl resource selection was influenced by the amount of these vegetation types within the Lake St. Clair region regardless of who was the managing entity.

Model (4) was the full model of landscape composition by ownership that included the area of different vegetation types categorized by ownership. This model would be appropriate if waterfowl resource selection was influenced by the amount of these resource with the Lake St. Clair region and management differences based on ownership.

Supporting-Table 3. Reference number for candidate models associated with Markov Chain Monte Carlo (MCMC) settings to investigate adult female mallard resource selection (A) prior to the hunting season, (B) the first of the hunting season and (C) second half of the hunting season and (D) post hunting season during the 2014–15 and 2015–16 monitoring periods of adult female mallards in Lake St. Clair region.

| **A. Preseason** |  |  |  |  |  | **B. First Half of the Hunting Season** |  |  |  |  |
| --- | --- | --- | --- | --- | --- | --- | --- | --- | --- | --- |
| Diel Period | Model | Iterations | Burn-in | Thinning |  | Diel Period | Model | Iterations | Burn-in | Thinning |
| Diurnal | 1 | NA | NA | NA |  | Diurnal | 1 | NA | NA | NA |
|  | 2 | 125,000 | 25,000 | 1 |  |  | 2 | 100,000 | 5,000 | 1 |
|  | 3 | 100,000 | 5,000 | 1 |  |  | 3 | 100,000 | 5,000 | 1 |
|  | 4 | 100,000 | 5,000 | 1 |  |  | 4 | 125,000 | 25,000 | 1 |
| Nocturnal | 1 | NA | NA | NA |  | Nocturnal | 1 | NA | NA | NA |
|  | 2 | 100,000 | 5,000 | 1 |  |  | 2 | 100,000 | 5,000 | 1 |
|  | 3 | 100,000 | 5,000 | 1 |  |  | 3 | 100,000 | 5,000 | 1 |
|  | 4 | 100,000 | 5,000 | 1 |  |  | 4 | 100,000 | 5,000 | 1 |
|  |  |  |  |  |  |  |  |  |  |  |
| **C. Second Half of the Hunting Season** |  |  |  |  |  | **D. Post Season** |  |  |  |  |
| Diel Period | Model | Iterations | Burn-in | Thinning |  | Diel Period | Model | Iterations | Burn-in | Thinning |
| Diurnal | 1 | NA | NA | NA |  | Diurnal | 1 | NA | NA | NA |
|  | 2 | 100,000 | 5,000 | 1 |  |  | 2 | 100,000 | 5,000 | 1 |
|  | 3 | 100,000 | 5,000 | 1 |  |  | 3 | 100,000 | 5,000 | 1 |
|  | 4 | 100,000 | 5,000 | 1 |  |  | 4 | 100,000 | 5,000 | 1 |
| Nocturnal | 1 | NA | NA | NA |  | Nocturnal | 1 | NA | NA | NA |
|  | 2 | 100,000 | 5,000 | 1 |  |  | 2 | 100,000 | 5,000 | 1 |
|  | 3 | 100,000 | 5,000 | 1 |  |  | 3 | 100,000 | 5,000 | 1 |
|  | 4 | 100,000 | 5,000 | 1 |  |  | 4 | 100,000 | 5,000 | 1 |

Supporting-Table 4. Deviance information criterion values for all resource selection models during the PRE hunting season, FIRST half of the hunting season, SECOND half of the hunting season, and POST hunting season seasons and for both diurnal and nocturnal diel periods.

|  |  |  |  |  |  |  |  |  |
| --- | --- | --- | --- | --- | --- | --- | --- | --- |
| Deviance Information Criterion Values | | | | | | | | |
|  | Pre | | First | | Second | | Post | |
| Model | Diurnal | Nocturnal | Diurnal | Nocturnal | Diurnal | Nocturnal | Diurnal | Nocturnal |
| 4 | 7659.5 | 3666.5 | 4832.3 | 5539.6 | 5281.2 | 6621.5 | 1276.3 | 988.4 |
| 3 | 9297.8 | 5324.5 | 6363.2 | 6807.2 | 6027.2 | 7135.5 | 1338.4 | 1098.3 |
| 2 | 11739.0 | 5837.5 | 9519.8 | 9928.7 | 8158.8 | 10254.4 | 1621.1 | 1381.1 |
| 1 | 14581.7 | 6519.4 | 18512.9 | 15996.1 | 13100.8 | 13372.7 | 2047.0 | 1684.2 |

Supporting-Table 5. Population selection parameter estimates and standard errors for the top discrete-choice models that investigated resource selection strategies for adult female mallards PRIOR to the hunting season in the Lake St. Clair region during the 2014–15 and 2015–16 monitoring years. A. Diurnal B. Nocturnal

A.

| Variable | Diel Period | Population selection parameter estimate | Standard deviation | 95% Credible interval lower bound | 95% Credible Interval upper bound |
| --- | --- | --- | --- | --- | --- |
| CWS-MARSH | DAY | 0.905 | 0.086 | 0.734 | 1.074 |
| MICH-DNR |  | 0.743 | 0.307 | 0.068 | 1.289 |
| MICH-WATER |  | -0.499 | 0.448 | -1.465 | 0.296 |
| PRI-AGRI |  | 1.87 | 0.507 | 0.867 | 2.86 |
| PRI-FLAG |  | 0.662 | 0.073 | 0.516 | 0.803 |
| PRI-MARSH |  | 0.79 | 0.128 | 0.537 | 1.041 |
| PRI-SUPP |  | 1.298 | 0.121 | 1.056 | 1.533 |
| PRI-WATER |  | 1.574 | 0.137 | 1.302 | 1.837 |
| PUB-MARSH |  | -1.237 | 0.400 | -2.103 | -0.531 |
| PUB-WATER |  | 2.785 | 0.487 | 1.844 | 3.77 |
| WAL-AGRI |  | 0.313 | 0.262 | -0.264 | 0.758 |
| WAL-MARSH |  | 0.162 | 0.396 | -0.675 | 0.881 |
| WAL-WATER |  | -0.036 | 0.229 | -0.529 | 0.377 |

B.

| Variable | Diel Period | Population selection parameter estimate | Standard deviation | 95% Credible interval lower bound | 95% Credible Interval Upper bound |
| --- | --- | --- | --- | --- | --- |
| CWS-MARSH | NIGHT | 0.024 | 0.232 | -0.468 | 0.442 |
| MICH-DNR |  | 0.661 | 0.298 | 0.046 | 1.235 |
| MICH-WATER |  | -0.213 | 0.433 | -1.15 | 0.557 |
| PRI-AGRI |  | -0.066 | 0.701 | -1.423 | 1.328 |
| PRI-FLAG |  | 0.618 | 0.093 | 0.426 | 0.795 |
| PRI-MARSH |  | 0.948 | 0.147 | 0.66 | 1.236 |
| PRI-SUPP |  | 0.578 | 0.148 | 0.283 | 0.864 |
| PRI-WATER |  | 1.823 | 0.145 | 1.542 | 2.113 |
| PUB-MARSH |  | 0.535 | 0.225 | 0.075 | 0.954 |
| PUB-WATER |  | 2.595 | 0.714 | 1.236 | 4.031 |
| WAL-AGRI |  | -0.427 | 0.463 | -1.431 | 0.375 |
| WAL-MARSH |  | 0.031 | 0.381 | -0.756 | 0.743 |
| WAL-WATER |  | -0.278 | 0.299 | -0.903 | 0.261 |

Supporting-Table 6. Population selection parameter estimates and standard errors for the top discrete-choice models that investigated resource selection strategies for adult female mallards FIRST half to the hunting season in the Lake St. Clair region during the 2014–15 and 2015–16 monitoring years. A. Diurnal B. Nocturnal

A.

| Variable | Diel Period | Population selection parameter estimate | Standard deviation | 95% Credible interval lower bound | 95% Credible interval upper bound |
| --- | --- | --- | --- | --- | --- |
| CWS-MARSH | DAY | 1.215 | 0.190 | 0.842 | 1.593 |
| MICH-DNR |  | 2.202 | 0.436 | 1.346 | 3.058 |
| MICH-WATER |  | -0.558 | 0.620 | -1.860 | 0.576 |
| PRI-AGRI |  | 2.126 | 0.951 | 0.215 | 3.967 |
| PRI-FLAG |  | 1.367 | 0.290 | 0.790 | 1.923 |
| PRI-MARSH |  | 1.627 | 0.317 | 1.000 | 2.240 |
| PRI-SUPP |  | 1.668 | 0.206 | 1.269 | 2.079 |
| PRI-WATER |  | 1.193 | 0.304 | 0.582 | 1.783 |
| PUB-MARSH |  | -3.109 | 0.728 | -4.643 | -1.763 |
| PUB-WATER |  | 0.223 | 0.777 | -1.268 | 1.769 |
| WAL-AGRI |  | -0.594 | 0.512 | -1.680 | 0.328 |
| WAL-MARSH |  | 1.304 | 0.469 | 0.309 | 2.165 |
| WAL-WATER |  | -1.248 | 0.450 | -2.176 | -0.410 |

B.

| Variable | Diel Period | Population selection parameter estimate | Standard deviation | 95% Credible interval lower bound | 95% Credible Interval Upper bound |
| --- | --- | --- | --- | --- | --- |
| CWS-MARSH | NIGHT | 0.696 | 0.158 | 0.381 | 0.998 |
| MICH-DNR |  | 1.714 | 0.395 | 0.904 | 2.468 |
| MICH-WATER |  | 0.184 | 0.393 | -0.610 | 0.939 |
| PRI-AGRI |  | -0.835 | 0.688 | -2.218 | 0.486 |
| PRI-FLAG |  | 1.317 | 0.145 | 1.034 | 1.606 |
| PRI-MARSH |  | 0.960 | 0.215 | 0.524 | 1.375 |
| PRI-SUPP |  | 0.984 | 0.167 | 0.650 | 1.309 |
| PRI-WATER |  | 0.683 | 0.244 | 0.197 | 1.156 |
| PUB-MARSH |  | -1.646 | 0.452 | -2.600 | -0.829 |
| PUB-WATER |  | -0.822 | 0.729 | -2.266 | 0.604 |
| WAL-AGRI |  | -1.272 | 0.536 | -2.422 | -0.323 |
| WAL-MARSH |  | 0.595 | 0.426 | -0.311 | 1.371 |
| WAL-WATER |  | -1.340 | 0.442 | -2.261 | -0.537 |

Supporting-Table 7. Population selection parameter estimates and standard errors for the top discrete-choice models that investigated resource selection strategies for adult female mallards SECOND half the hunting season in the Lake St. Clair region during the 2014–15 and 2015–16 monitoring years. A. Diurnal B. Nocturnal.

A.

| Variable | Diel Period | Population selection parameter estimate | Standard deviation | 95% Credible interval lower bound | 95% Credible Interval Upper bound |
| --- | --- | --- | --- | --- | --- |
| CWS-MARSH | DAY | 0.935 | 0.153 | 0.629 | 1.239 |
| MICH-DNR |  | 1.943 | 0.439 | 1.053 | 2.789 |
| MICH-WATER |  | 0.535 | 0.581 | -0.686 | 1.606 |
| PRI-AGRI |  | 0.235 | 0.615 | -0.983 | 1.433 |
| PRI-FLAG |  | 0.821 | 0.129 | 0.562 | 1.071 |
| PRI-MARSH |  | 0.449 | 0.230 | -0.018 | 0.892 |
| PRI-SUPP |  | 1.066 | 0.123 | 0.83 | 1.313 |
| PRI-WATER |  | 0.602 | 0.221 | 0.155 | 1.022 |
| PUB-MARSH |  | -1.844 | 0.464 | -2.846 | -1.012 |
| PUB-WATER |  | 0.483 | 0.524 | -0.539 | 1.522 |
| WAL-AGRI |  | -0.854 | 0.494 | -1.931 | -0.003 |
| WAL-MARSH |  | 0.515 | 0.385 | -0.281 | 1.231 |
| WAL-WATER |  | -1.035 | 0.371 | -1.823 | -0.362 |

B.

| Variable | Diel Period | Population selection parameter estimate | Standard deviation | 95% Credible interval lower bound | 95% Credible Interval Upper bound |
| --- | --- | --- | --- | --- | --- |
| CWS-MARSH | NIGHT | 0.704 | 0.124 | 0.458 | 0.949 |
| MICH-DNR |  | 1.765 | 0.351 | 1.043 | 2.43 |
| MICH-WATER |  | 1.275 | 0.352 | 0.557 | 1.952 |
| PRI-AGRI |  | -0.817 | 0.568 | -1.934 | 0.299 |
| PRI-FLAG |  | 0.591 | 0.132 | 0.322 | 0.845 |
| PRI-MARSH |  | 0.142 | 0.158 | -0.188 | 0.435 |
| PRI-SUPP |  | 1.351 | 0.114 | 1.121 | 1.572 |
| PRI-WATER |  | 0.518 | 0.201 | 0.118 | 0.905 |
| PUB-MARSH |  | -0.241 | 0.253 | -0.771 | 0.222 |
| PUB-WATER |  | 1.253 | 0.486 | 0.322 | 2.227 |
| WAL-AGRI |  | -0.719 | 0.421 | -1.638 | 0.001 |
| WAL-MARSH |  | -0.011 | 0.373 | -0.79 | 0.674 |
| WAL-WATER |  | -0.86 | 0.330 | -1.55 | -0.256 |

Supporting- Table 8. Population selection parameter estimates and standard errors for the top discrete-choice models that investigated resource selection strategies for adult female mallards POST hunting season in the Lake St. Clair region during the 2014–15 and 2015–16 monitoring years. A. Diurnal B. Nocturnal

A.

| Variable | Diel Period | Population selection parameter estimate | Standard deviation | 95% Credible interval lower bound | 95% Credible Interval Upper bound |
| --- | --- | --- | --- | --- | --- |
| CWS-MARSH | DAY | 0.92 | 0.181 | 0.558 | 1.275 |
| MICH-DNR |  | 1.35 | 0.618 | 0.086 | 2.543 |
| MICH-WATER |  | 1.597 | 0.768 | 0.127 | 3.133 |
| PRI-AGRI |  | 1.234 | 0.798 | -0.314 | 2.818 |
| PRI-FLAG |  | 0.335 | 0.144 | 0.048 | 0.621 |
| PRI-MARSH |  | 0.026 | 0.281 | -0.563 | 0.54 |
| PRI-SUPP |  | 1.582 | 0.177 | 1.233 | 1.929 |
| PRI-WATER |  | 1.11 | 0.236 | 0.635 | 1.566 |
| PUB-MARSH |  | 0.089 | 0.360 | -0.684 | 0.742 |
| PUB-WATER |  | 2.203 | 0.782 | 0.699 | 3.768 |
| WAL-AGRI |  | 1.103 | 0.413 | 0.227 | 1.876 |
| WAL-MARSH |  | 0.427 | 0.531 | -0.679 | 1.415 |
| WAL-WATER |  | -0.289 | 0.486 | -1.333 | 0.57 |

B.

| Variable | Diel Period | Population selection parameter estimate | Standard deviation | 95% Credible interval lower bound | 95% Credible Interval Upper bound |
| --- | --- | --- | --- | --- | --- |
| CWS-MARSH | NIGHT | 0.841 | 0.232 | 0.357 | 1.27 |
| MICH-DNR |  | 0.899 | 0.630 | -0.388 | 2.099 |
| MICH-WATER |  | 1.994 | 0.880 | 0.349 | 3.813 |
| PRI-AGRI |  | -3.526 | 1.115 | -5.76 | -1.388 |
| PRI-FLAG |  | 0.626 | 0.228 | 0.153 | 1.057 |
| PRI-MARSH |  | 0.475 | 0.320 | -0.183 | 1.078 |
| PRI-SUPP |  | 1.56 | 0.391 | 0.779 | 2.322 |
| PRI-WATER |  | 0.854 | 0.276 | 0.282 | 1.366 |
| PUB-MARSH |  | 0.498 | 0.392 | -0.296 | 1.263 |
| PUB-WATER |  | 3.295 | 0.969 | 1.455 | 5.239 |
| WAL-AGRI |  | 0.75 | 0.542 | -0.412 | 1.728 |
| WAL-MARSH |  | -0.56 | 0.667 | -1.939 | 0.672 |
| WAL-WATER |  | -0.116 | 0.541 | -1.267 | 0.858 |
|  |  |  |  |  |  |
