# Supplementary figures and images for: Mallard resource selection trade‐offs in a heterogeneous environment during autumn and winter
Source: Ecol Evol. 2019 Feb 6;9(4):1798–808. doi: 10.1002/ece3.4864 (PMC6392399; doi:10.1002/ece3.4864)

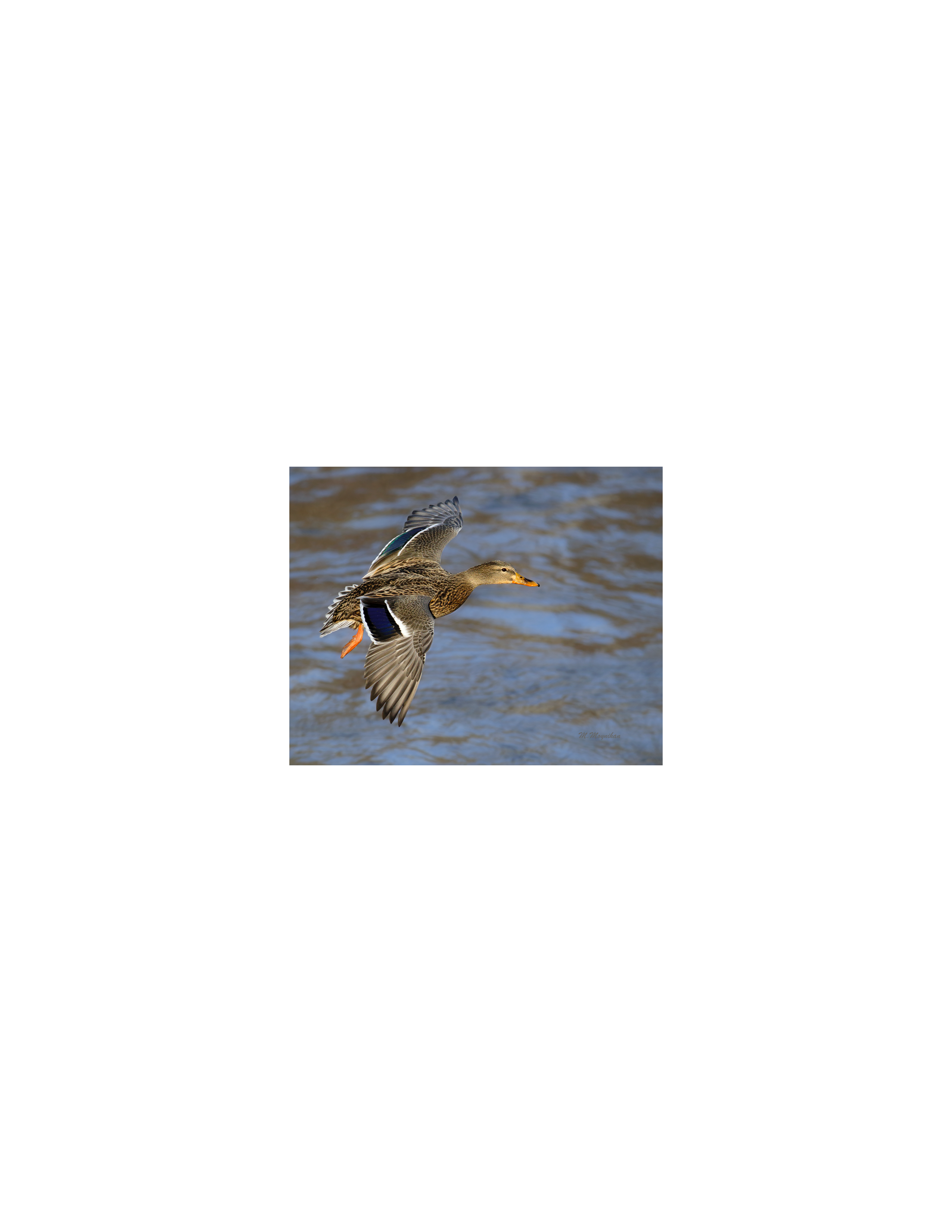

Supplement: Supplementary file 2 [file ECE3-9-1798-s002.tiff]
